# A case of reinfection with a different variant of SARS-CoV-2: case report

**DOI:** 10.1186/s43162-023-00194-4

**Published:** 2023-02-08

**Authors:** Nagashige Shimada, Masahiro Shinoda, Hiroaki Takei, Yuto Yoshida, Masashi Nishimura, Mio Kousaka, Miwa Morikawa, Takashi Sato, Hiroto Matsuse, Masaharu Shinkai

**Affiliations:** 1 Department of Respiratory Medicine, Tokyo Shinagawa Hospital, Tokyo, Japan; 2 Division of Respiratory Medicine, Department of Internal Medicine, Toho University Ohashi Medical Center, Tokyo, Japan

**Keywords:** SARS-CoV-2, Reinfection, Variant, N501Y, Alpha variant, E484K

## Abstract

**Background:**

Coronavirus disease 2019 (COVID-19) was previously thought to have a low reinfection rate, but there are concerns that the reinfection rate will increase with the emergence and spread of mutant variants. This report describes the case of a 36-year-old, non-immunosuppressed man who was infected twice by two different variants of COVID-19 within a relatively short period.

**Case presentation:**

A 36-year-old Japanese man with no comorbidities was infected with the E484K variant (R.1 lineage) of severe acute respiratory syndrome coronavirus 2 (SARS-CoV-2). Symptoms were mild and improved with symptomatic treatment alone. About four months later he presented to another outpatient department with high fever and headache. We diagnosed him as infected with the Alpha variant (B.1.1.7) of SARS-CoV-2 based on SARS-CoV-2 real-time reverse transcription polymerase chain reaction testing (RT-PCR). The patient was hospitalized with high fever. The patient received treatment in the form of anti-inflammatory therapy with corticosteroid and antibacterial chemotherapy. The patient improved without developing severe disease.

**Conclusion:**

Concerns have been raised that the reinfection rate of COVID-19 will increase with the emergence of mutant variants. Particularly in mild cases, adequate amounts of neutralizing antibodies may not be produced, and reinfection may thus occur. Continued attention to sufficient infection control is thus essential.

## Introduction

Defensive immunity after coronavirus disease 2019 (COVID-19) caused by severe acute respiratory syndrome coronavirus 2 (SARS-CoV-2) infection has not been fully elucidated. The emergence of variants is considered to increase the risk of reinfection [1]. While reinfection has been reported [2], little data regarding reinfection has been accumulated. To the best of our knowledge, no cases of COVID-19 reinfection caused by multiple variants have previously been reported in detail. We describe two distinct infective episodes of COVID-19 caused by different variants occurring within a relatively short period in the same individual in Japan.

## Case report

**Fig. 1:**
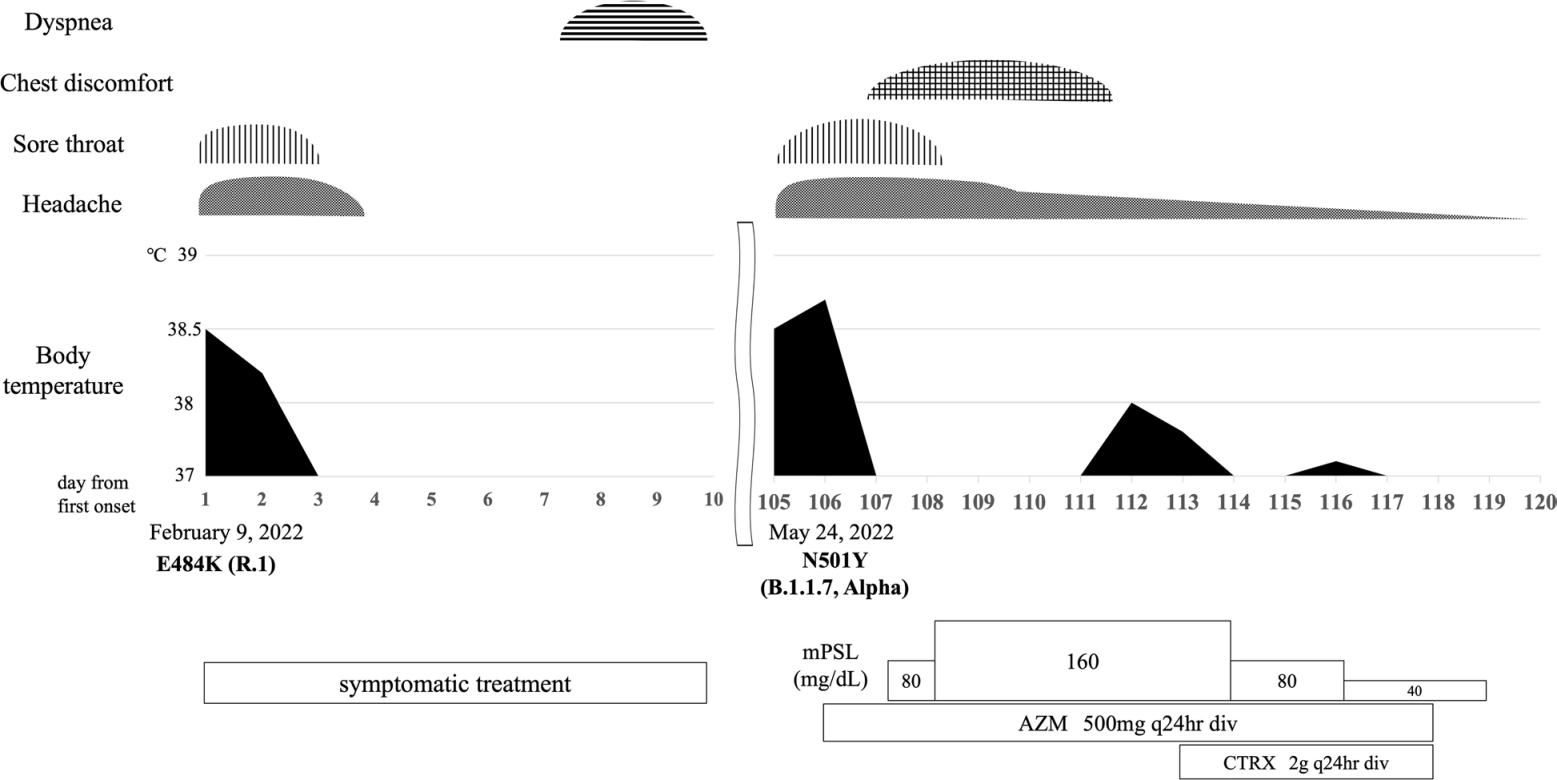
The course of symptoms and treatments from infection with the E484K variant to infection with the N501Y variant. The severity was mild in both the initial infection (E484K variant) and the reinfection (N 501Y variant). In the initial infection, he was relieved in about 3 to 4 days with only symptomatic treatment, but in the reinfection, his fever was bimodal, and he had persistent symptoms. Abbreviations: methylprednisolone, mPSL; azithromycin, AZM; ceftriaxone, CTRX

A 36-year-old Japanese man with no comorbidities was diagnosed with COVID-19 caused by E484K variant (R.1 lineage) on February 9, 2021 (The machine we used for the SARS-CoV-2 real-time RT-PCR testing was a Thermo Fisher Scientific QuantStudio5). He smoked one pack of cigarettes daily, drank socially, and worked in a call center. At that time, he presented to our outpatient department with fever ranging from 37.5 °C to 38 °C, headache, and sore throat. His height was 173 cm, his body weight was 71 kg, and his BMI was 23.7. His consciousness was clear, body temperature was 36.0 °C, blood pressure was 122/72 mmHg, pulse rate was 84/min, respiratory rate 18/min, and SpO2 was 98% (ambient air). Breath sounds were clear on chest auscultation without asymmetry and adventitious sound. No other physical findings were found. Blood tests showed mild increases in C-reactive protein (CRP) and ferritin. The white blood cell count was slightly increased, and the lymphocyte fraction was decreased. No increase was evident in lactate dehydrogenase (LDH), and no abnormalities were seen in coagulation tests (Table 1). X-ray and computed tomography of the chest showed no abnormalities. SARS-CoV-2 real-time RT-PCR testing yielded positive results for the E484K variant, and negative results for the N501Y variant (cycle threshold [Ct] value, 33). Symptoms were mild and resolved within 21 days with symptomatic treatment alone (Fig. 1).

**Table 1:** Comparison of laboratory findings at diagnosis at the time of primary infection and reinfection

Blood count				Biochemistry				Coagulation			
	Primary infection	Reinfection			Primary infection	Reinfection			Primary infection	Reinfectio*n*	
WBC	11,000	5300	/μl	T-Bil	0.32	0.3	mg/dl	PT-INR	1.12	1.1	
Neut	78.4	70.7	%	AST	16	24	IU/l	APTT	30.9	36.4	s
Lymph	16	20.5	%	ALT	21	31	IU/l	Fibrinogen	368	273	mg/dl
Mono	5.1	8.4	%	LDH	123	141	IU/l	FDP	< 2.5	2.5	μg/ml
Eosin	0.3	0	%	BUN	16.5	13.4	mg/dl	D-dimer	0.2	0.8	μg/ml
Baso	0.2	0.4	%	Cr	0.88	1.04	mg/dl				
RBC	455	475	× 10^4^/μl	Na	141	139	mEq/l				
Hb	13.9	13.9	g/dl	K	4.5	4	mEq/l				
Hct	40.9	42.9	%	Cl	103	102	mEq/l				
PLT	22.6	16.6	× 10^4^/μl	CRP	0.95	0.51	g/dl				
				Ferritin	334	294	ng/ml				
				sIL-2R	–	252	U/ml				
				KL-6	–	121	U/ml				
				SP-A	–	16.9	ng/ml				
				SP-D	–	15	ng/ml

On May 24, 2021, he presented to another outpatient department with high fever and headache and was again diagnosed with alpha variant (B.1.1.7) based on SARS-CoV-2 real-time RT-PCR testing. While he had no recent travel history, the patient had eaten with friends on several occasions before this onset, suggesting a possible exposure. He was admitted to our hospital 1 day after symptom onset. His consciousness was clear, body temperature was 38.0 °C, blood pressure was 94/68 mmHg, pulse rate was 81/min, respiratory rate 16/min, and SpO2 was 98% (ambient air). Breath sounds were clear on chest auscultation without asymmetry and adventitious sound. No other physical findings were found. Physical examination on admission showed a body temperature of 38.0 °C, but otherwise normal results. Blood tests showed mild increases in CRP and ferritin. No increase was seen in white blood cell count, but the lymphocyte fraction was decreased. X-ray and computed tomography of the chest again showed no abnormalities. No abnormalities were seen in coagulation tests (Table 1). SARS-CoV-2 real-time RT-PCR testing yielded positive results for the N501Y variant and negative results for the E484K variant (Ct value, 14). We diagnosed COVID-19 reinfection with a different SARS-CoV-2 variant (Alpha variant) because the Ct value was low, and the variants recognized differed. Although disease severity was mild, the patient exhibited persistent high-grade fevers (maximum temperature 38.2 °C) and was treated with methylprednisolone (mPSL; 80 mg/day on day 2 of hospitalization, 160 mg/day on day 3–8) and azithromycin for their anti-viral properties (500 mg/day from day 1). He did not receive antiviral treatment because he was deemed to not be high risk for progression to severe disease. Although high fever and headache improved with anti-inflammatory treatment, fever reappeared from day 7 of illness and CRP again increased. We considered the possibility of secondary infection first and exacerbation of inflammation by SARS-CoV2. He received ceftriaxone (2 g/day on day 8–12) and decreased the dose of mPSL (80 mg/day on day 9–10, 40 mg/day on day 11–13). We initially scheduled to have him receive azithromycin for about 7 days in anticipation of antiviral effects but continued in anticipation of anti-inflammatory cytokine effects and immunomodulatory effects. After that, fever improved immediately. He experienced tingling in the chest and difficulty breathing during hospitalization, but no shadows were apparent on chest X-rays. The patient was discharged on day 15 after admission (Fig. 1). To date, no sequelae have been observed.

## Discussion and conclusion

In this case, a young male with no relevant medical history was re-infected with two different variants of SARS-CoV-2 within a relatively short period of about four months.

**Fig. 2:**
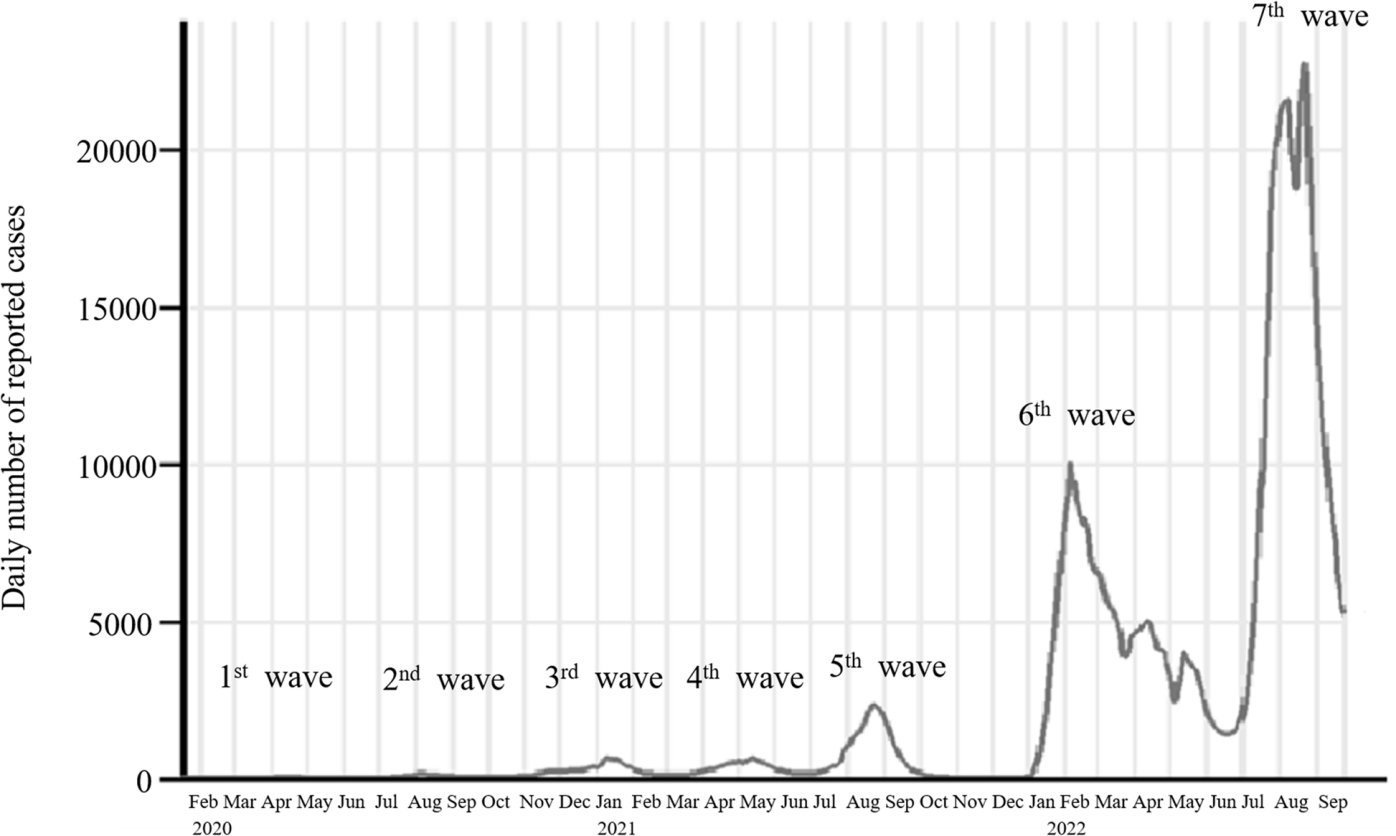
Trends in the daily number of COVID-19 cases reported in Japan. The number of COVID-19 cases has increased remarkably in the 5th, 6th, and 7th waves. Chart elaborated at https://www.iancampbell.co.uk/covid-19.php with data from johns Hopkins university. Last accessed 27 September 2022

COVID-19 was initially identified in December 2019 and since then has made its presence known globally. The first case was reported in Japan on 15 January 2020 in a man who had returned from Wuhan, Hubei Province, People’s Republic of China. Since then, seventh epidemic waves have been observed in Japan (Fig. 2) [3, 4]. Since around the end of 2020, Alpha variant (B.1.1.7, UK variant; mainly comprising the N501Y variant) and Beta variant (B.1.351) or Gamma variant (P.1, South African variant, or Brazilian variant, respectively; mainly comprising N501Y and E484K variants) have been reported worldwide [5]. Likewise, in Japan, On 25 December 2020, Alpha variant was first detected in airport quarantine in returnees from the UK. The proportion of individuals infected with Alpha variant has increased since January 2021 and became as the fourth wave in Japan [6]. Delta variant (Indian variant, mainly comprising the L452R and E484Q variants) was first detected in Indian in October 2020, and detected in the quarantine of Japan on 28 March 2021, and the domestic case was first seen on 20 April 2021, and it became a pandemic as the fifth wave [7].

Alpha variant has a 43–90% higher adequate reproduction number than the conventional strain [8] and has been reported to increase the risk of death by 55% [9]. In vitro, neutralization assays on convalescent and vaccinated sera have revealed that Alpha variant required a higher serum concentration to achieve neutralization. However, no evidence suggested that Alpha variant could evade neutralization by serum raised to earlier SARS-CoV-2 strains or vaccines [10].

Before the epidemic of variants, a meta-analysis of COVID-19 reinfection by Arafkas M, et al. pointed out that the frequency of COVID-19 reinfection was low [11]. After infection with COVID-19, neutralizing antibodies are reportedly maintained for 6 months, and most mild cases also displayed neutralizing antibodies [12]. The amounts of neutralizing antibodies among individuals who have recovered from COVD-19 were reportedly significantly reduced, but the quality of neutralization was maintained, and a certain effect on variants was noted [13]. However, Vincent L, et al. reported that neutralizing antibody titers are high in severe cases but heterogeneous in mild cases, suggesting that adequate amounts of neutralizing antibodies may not be produced [14]. Reinfection with mutant variants such as Alpha variant [15] and E484K variant (P.2 lineage) [16] has been described in patients without immunosuppression, but details such as frequency were unclear. In the reported cases, the initial infection was mild. Since the disease, in this case, was mild at initial infection, enough neutralizing antibodies were presumably not produced, and a mutant variant with stronger infectivity caused reinfection.

R.1 lineage containing only the E484K variant was first detected in Japan in November 2020 and partially prevalent in the third wave from October 2020 to February 2021 [17]. The E484K variant was reported as an escape variant from a monoclonal antibody that neutralizes SARS-CoV-2 [18]. According to in vitro data, the E484K variant was an escape variant from convalescent plasma, and in the presence of antibodies against SARS-CoV2 in convalescent plasma, cell infection by viruses with the E484K variant was difficult to suppress [19]. Concerns have been raised that the E484K variant can reinfect individuals previously infected with COVID-19 [20], but clinical evidence for this has remained lacking. In addition, no information has been published on the production of neutralizing antibodies after infection with the E484K variant.

Theta variant (P.3, the Philippine variant; mainly comprising the N501Y and E484K variants) was detected in those who returned from the Philippines on February 25, 2021 [21]. This variant did not cause a significant epidemic in Japan. From around February 2022, B.1.1.529 (Omicron variant), which has higher transmissibility, viral infectivity, and immune evasion potential, became prevalent in Japan [4], and it became a pandemic as the sixth wave. After that, due to the emergence of subvariant, it became a pandemic as the seventh wave (Fig. 2). Reinfection with phylogenetically distinct omicron variants has been reported [22]. In vitro, BA.5 may escape neutralizing antibodies acquired by infection with BA.1 and BA.2 [23, 24]. Therefore, the risk of reinfection is concerned, and strengthening immunity such as COVID-19 vaccine booster shots and vaccine shots for variants are becoming more critical.

In conclusion, the risk of reinfection was basically low before the variant epidemic, but the appearance of variants may increase the reinfection rate. Particularly in mild cases, neutralizing antibodies may not be produced in adequate amounts, allowing reinfection to occur. Continued attention to sufficient infection control is thus essential.

## Data Availability

The datasets generated during and/or analyzed during the current study are available from the corresponding author on reasonable request.

## Abbreviations

AZM: Azithromycin
COVID-19: Coronavirus disease 2019
CRP: C-reactive protein
CTRX: Ceftriaxone
Ct value: Cycle threshold value
LDH: Lactate dehydrogenase
mPSL: Methylprednisolone
RT-PCR: Reverse-transcription polymerase chain reaction
SARS-CoV-2: Severe acute respiratory syndrome coronavirus 2
